# Assessing the Consequences of Stigma for Tuberculosis Patients in Urban Zambia

**DOI:** 10.1371/journal.pone.0119861

**Published:** 2015-03-25

**Authors:** Anne Lia Cremers, Myrthe Manon de Laat, Nathan Kapata, Rene Gerrets, Kerstin Klipstein-Grobusch, Martin Peter Grobusch

**Affiliations:** 1 Center of Tropical Medicine and Travel Medicine, Department of Infectious Diseases, Division of Internal Medicine, Academic Medical Center, University of Amsterdam, Amsterdam, The Netherlands; 2 Faculty of Social and Behavioural Science, Department of Sociology and Anthropology, University of Amsterdam, Amsterdam, The Netherlands; 3 The National TB/Leprosy Control Programme, Lusaka, Zambia; 4 University of Zambia—University College London (UNZA-UCL) program, Lusaka, Zambia; 5 Julius Global Health, Julius Center for Health Sciences and Primary Care, University Medical Center Utrecht, Utrecht, The Netherlands; 6 Division of Epidemiology, School of Public Health, Faculty of Health Sciences, University of the Witwatersrand, Johannesburg, South Africa; University of Missouri-Kansas City, UNITED STATES

## Abstract

**Background:**

Stigma is one of the many factors hindering tuberculosis (TB) control by negatively affecting hospital delay and treatment compliance. In Zambia, the morbidity and mortality due to TB remains high, despite extended public health attempts to control the epidemic and to diminish stigma.

**Study Aim:**

To enhance understanding of TB-related stigmatizing perceptions and to describe TB patients’ experiences of stigma in order to point out recommendations to improve TB policy.

**Methods:**

We conducted a mixed method study at Kanyama clinic and surrounding areas, in Lusaka, Zambia; structured interviews with 300 TB patients, multiple in-depth interviews with 30 TB patients and 10 biomedical health workers, 3 focus group discussions with TB patients and treatment supporters, complemented by participant observation and policy analysis of the TB control program. Predictors of stigma were identified by use of multivariate regression analyses; qualitative analysis of the in-depth interviews, focus group discussions and participant observation was used for triangulation of the study findings.

**Results:**

We focused on the 138/300 patients that described TB-related perceptions and attitudes, of whom 113 (82%) reported stigma. Stigma provoking TB conceptions were associated with human immunodeficiency virus (HIV)-infection, alleged immoral behaviour, (perceived) incurability, and (traditional) myths about TB aetiology. Consequences of stigma prevailed both among children and adults and included low self-esteem, insults, ridicule, discrimination, social exclusion, and isolation leading to a decreased quality of life and social status, non-disclosure, and/or difficulties with treatment compliance and adherence. Women had significantly more stigma-related problems than men.

**Conclusions:**

The findings illustrate that many TB patients faced stigma-related issues, often hindering effective TB control and suggesting that current efforts to reduce stigma are not yet optimal. The content and implementation of sensitization programs should be improved and more emphasis needs to be placed on women and children.

## Introduction

Alongside biological, economic, and cultural barriers to effective tuberculosis (TB) control, stigma constitutes one of the major social factors causing hospital delay and hindering compliance among TB patients [1,2]. Various studies conducted in different African settings have exposed negative attitudes towards TB patients and/or described the subsequent consequences. Oftentimes attitudes can be explained by local believes and knowledge of TB transmission, such as shared use of eating utensils [3], hereditary factors [3–5], sexual intercourse [6–8], bewitchment [3,5,9], smoking [3,4,7,8], heavy labour [3–5,7], human immunodeficiency virus (HIV)-infection [9–12], and poverty [13]. These perceptions lead to shame [12,14], fear of physical contact among community members [10], affected marriage prospects [15], social isolation [9,10,14], and discrimination [16]. The extensive systematic review of Chang et al (2014) describes that consequences of stigma hinder, or even adversely influence, efforts to stimulate treatment compliance and reduce delays in diagnosis and treatment worldwide [17]. Moreover, stigma impedes the application of preventive measures such as coughing-hygiene and good ventilation at home resulting in increased transmission risk, severe morbidity and mortality and increased development of multi-drug resistance (MDR-TB), thus undermining successful TB control [12,18,19].

Stigma is often explained as a discrediting attribute leading to an impairment of social status and position, rejection and/or exclusion [20]. In addition, stigma is seen as a key factor in the production and reproduction of power structures, causing devaluation of certain social groups or individuals, thus aiding social inequality [20–22]. Stigma can be differentiated into three main sub-categories: *experienced stigma* (the experience of exclusion and/or discrimination), *anticipated stigma* (the perception, expectation and/or fear of stigma), and *internalized stigma* (a loss of self-esteem, dignity, fear and/or shame) [1,18]. Since these sub-categories elicit the diversely layered struggles associated with stigma, we have applied those in this study. Taking into consideration that stigma varies from culture to culture [22,23], we will address the context of stigmatizing actors, local values and ideology, political organization and economic system of the society from which stigma arises [21,23]. Moreover, we will critically evaluate how the Zambian National TB Program (NTP) addresses TB-related stigma and whether the program plays a role in the (re)production of stigma.

The importance of addressing stigma related to TB is illustrated by the fact that this disease is one of the major causes of death worldwide. Zambia ranks 29^th^ among the world’s top TB countries identified by the World Health Organisation (WHO) having 427/100.000 incident TB cases in the year 2012. HIV co-infection rate is 61% and MDR-TB prevails in 0.3% of new TB-cases and 8.1% of retreatment TB-cases. Males are more often affected by TB than females (Zambian male-to-female ratio: 1.7:1) [24]. Besides epidemiological factors, the NTP faces a wide variety of clinical, operational, and social challenges.

Stigma is still a low-priority issue in international TB control efforts [1], notwithstanding the numerous papers written on this topic [2,4,19,25–27]. However, in Zambia, little research has been performed on HIV/TB-related stigma [12] and an assessment of the scope, nature, and social consequences of specifically TB-related stigma in Zambia has not been published.

Therefore, we combined both qualitative and quantitative research methods [28] to investigate TB-related stigma in an urban health care setting in Lusaka, Zambia. The research aim is to enhance understanding of stigma and its effects on TB patients’ lives and the NTP to offer suggestions for improving health interventions. Secondly, our study may serve as a baseline for monitoring and evaluating potential future interventions and prevalence of stigma over time.

## Methods

This research is part of the broader TB patients’ Adherence and Compliance (TBAC)-study that took place from September 2013 until January 2014 in Kanyama (Lusaka, Zambia), an urban squatter settlement characterised by poverty and a high TB prevalence. In the Kanyama clinic, a modified version of WHO Direct Observed Therapy (DOT) policy to address tuberculosis is applied. The first two months, smear-positive and DR-TB patients receive medication daily at the clinic, and smear-negative patients once a week. After this period, patients collect TB-drugs every two weeks. Treatment takes several months and on average a patient is no longer infectious after two weeks of continuous drug-intake. Isoniazide Preventive Therapy (IPT) is offered for HIV-infected children who have close contact with TB patients.

Various (education) programs address TB-related stigma: treatment starts with a one-on-one talk to educate patients about TB. A treatment supporter is assigned to each patient for answering questions, family-sensitization, treatment encouragement, or finding patients who are lost to follow up (LTF). A household member of the patient gets involved in supervising treatment intake. In addition, a counsellor is available for TB patients and support groups are organised for people with TB-HIV co-infection. Treatment supporters organise twice a week sensitization programs in the community. They go from door to door to discuss TB and to hand out information brochures. Occasionally, they give a music and theatre show about TB. Additionally, the clinic allocates long-term TB nurses with expertise in TB, putative aetiologies, fear of transmission, and stigma-related struggles.

We used a mixed method design in a sequential explanatory model for which we first collected quantitative data and subsequently qualitative data to gain an in-depth understanding of statistical relationships and its context [16].

The four-month study period enabled us to conduct structured interviews with 300 (extra-) pulmonary TB patients undergoing Direct Observed Therapy at Kanyama clinic willing to participate in the study. Respondents under age eighteen were either interviewed with permission of their parent/guardian or their parent/guardian was interviewed. All 300 patients completed the structured interview. The sample size was considered to be sufficient to investigate the nine predictors of compliance for the TBAC study, considering a prevalence of defaulting patients of 30% of the overall study group [29] and the sample size recommendations for a logistic regression analysis [30] to investigate predictors with a 95% confidence interval. For this sub-study on stigma, we focused on the 138 patients who where identified to have experienced stigma, defined as having experienced negative or positive attitudes or perceptions regarding TB patients.

Additionally, we conducted qualitative research for which we randomly selected 30 patients of the study group (N = 300) for in-depth interviews of which six patients were younger than 20 years. Respondents under age eighteen were either interviewed with permission of their parent/guardian or their parent/guardian was interviewed.

Moreover, we approached ten biomedical health care providers (both nurses and treatment supporters of the TB department) for in-depth semi-structured interviews in the clinic. In addition, we organised three focus group discussions (FGD) in a secluded area of the clinic in order to enhance confidentiality. We randomly recruited ten treatment supporters for the first FGD and eight patients and two treatment supporters for both the second and third FGD. Participant observation was conducted at the clinic, during sensitization programs in the community, and in patients’ homes/neighbourhoods.

The structured interview contained questions regarding demographics, treatment history, bio-medical knowledge, and TB-related difficulties. Three questions were stigma-related: Do you feel shy/shame coming to the clinic? What do people in the place you live/in your neighbourhood think about TB? How do they compare HIV and TB? The questions were based on the literature [1, 18], screened by a Zambian medical doctor and the nurses of the TB corner in Kanyama clinic, and tested in a two-week pilot study. Subsequently some questions were adjusted, added or deleted to optimise the questionnaire for use in the current study. The interviewer fitted the responses to response-options using verbal and numeric labels. We allowed space for respondents to elaborate on their answers or to provide additional comments.

We visited 30 patients 1–3 times at their homes for in-depth, semi-structured interviews (1–2 hours each) extensively assessing stigma-related topics. The in-depth interviews with health workers focused on the functioning of the TB program and the challenges in their work. The FGDs addressed (1) childhood TB & parents, (2) problems of TB patients encountered in daily life, and (3) TB-related stigma. We used various techniques to foster an informal setting in which respondents felt free to talk, such as forming word clouds, theme ranking, making posters, and group presentations. All interviews and FGDs were conducted by a medical anthropologist and local research assistant in the local Zambian languages (English, Nyanja, Lhosi, Tonga, or Bemba) and transcribed into English.

We divided the 138 included patients in two groups: one comprising patients describing negative attitudes/perceptions (*stigma*), and another with patients describing supportive or positive responses from their social environment (*no stigma*). Furthermore, the group of stigmatized TB patients was subdivided into the above-mentioned subcategories experienced, anticipated and/or internalized stigma [1,18].

In order to identify factors that rendered a TB patient more vulnerable for stigma, we conducted univariate logistic regression analyses. Variables that had an association with stigma with a significance of p <0.1 were subsequently included in a multivariate regression model predicting stigma. We compared the sample (N = 30) undergoing multiple in-depth interviews with the overall study group (N = 300) regarding demographics (sex, age, education, marital status), treatment-related parameters (HIV co-infection, TB relapse, treatment duration, TB knowledge), and the percentage of people suffering from stigma applying Chi Square and T-tests. All analytic tests were performed using IBM SPSS statistics version 21.0 (IBM Corp, Armonk, NY).

Qualitative data analysis was conducted to explain, contextualize, and interpret quantitative outcomes. Using Qualitative Data Analysis and Research Software (ATLAS.ti, 7^th^ edition; Scientific Software Development GmbH, Berlin, Germany), we conducted thematic and content analysis for the in-depth interviews and FGDs. Transcripts were screened multiple times, coded into meaning units and categorized into broad themes [31]. Additionally, we analysed context, meaning and structures of identified codes and themes and explained statistical relationships found in quantitative analysis [32]. Some quotes of respondents were used to illustrate most important themes.

Ethical approval (HSSREC 02-08-13) for the study was obtained from the University of Zambia Biomedical Research Ethics Committee (UNZAREC). Written informed consent was obtained from all informants using UNZAREC-forms prior to inclusion in the study. For respondents under age eighteen we asked additional written informed consent from their parent/guardian. We guaranteed anonymity and confidentiality of given information by safe storage of data, usage of pseudonyms, and unidentifiable descriptions of patients throughout this article. Data were stored safely under lock and key. Only the main investigator had access to these data.’

## Results

Mean age of TB patients participating in the study (N = 300) was 33 years, ranging from 1 to 70 years and including 25 children and teenagers aged 1 to 19 years. Almost two thirds (64.3%) were male; 148 patients (49.3%) had a low education level (0–7 school years), and about half (49.0%) were co-infected with HIV (Table 1). The sample of 30 TB patients that was followed up for in-depth interviews did not significantly differ from the overall study group (N = 300) concerning study population characteristics (Table 2).

**Table 1:** Socio-demographic characteristics and tuberculosis-related parameters of TBAC study participants, Lusaka, Zambia.

Variable	All TB patients N = 300 (%)	Patients reporting TB-related perceptions & attitudes N = 138 (%)	Patients not reporting TB-related perceptions & attitudes N = 162 (%)	P-value
**Sex**				0.850
Male	193 (64.3%)	88 (63.8%)	105 (64.8%)	
Female	107 (35.7%)	50 (36.2%)	57 (35.2%)	
**Age (yrs)**				**0.030**
*Mean (SD* *)	33.3 (11.33)	31.8 (11.15)	34.6 (11.35)	
**Level of education**				0.061
Low (<8 yrs)	148 (49.3%)	60 (43.5%)	88 (54.3%)	
High (≥8 yrs)	152 (50.7%)	78 (56.5%)	74 (45.7%)	
**Marital Status**				0.207
Relationship	121 (40.3%)	1 (44.2%)	60 (37.0%)	
Single	179 (59.7%)	77 (55.8%)	102 (63.0%)	
**HIV co-infection**				0.451
Yes	147 (49.0%)	73 (52.9%	74 (45.7%)	
No	126 (42.0%)	54 (39.1%)	72 (44.4%)	
Unknown	27 (9.0%)	11 (8.0%)	16 (9.9%)	
**TB-relapse**				0.310
Yes	86 (29.0%)	36 (26.1%)	50 (31.4%)	
No	211 (71.0%)	102 (73.9%)	109 (68.6%)	
**Duration treatment (weeks)**				0.135
*Mean (SD)*	10.8 (8.33)	10.0 (7.95)	11.5 (8.60)	
**Knowledge of TB**				0.724
None	175 (58.3%)	82 (59.4%)	93 (57.4%)	
Some	125 (41.7%)	56 (40.6%)	69 (42.6%)	

*Standard Deviation

**Table 2:** Socio-demographic characteristics and tuberculosis-related parameters of in-depth sample of TBAC study, Lusaka, Zambia.

Variable	All N = 30 (%)	Stigma N = 18 (%)	No Stigma N = 12 (%)	P-value
**Sex**				**0.024**
Male	17 (56.7%)	7 (38.9%)	10 (83.3%)	
Female	13 (43.3%)	11 (61.1%)	2 (16.7%)	
**Age (yrs)**				0.442
*Mean (SD)*	31.0 (13.29)	29.5 (12.67)	33.3 (14.42)	
**Level of education**				0.879
Low (<8 yrs)	12 (40.0%)	7 (38.9%)	5 (41.7%)	
High (≥8 yrs)	18 (60.0%)	11 (61.1%)	7 (58.3%)	
**Marital Status**				0.060
Single	14 (46.7%)	11 (61.1%)	3 (25.0%)	
Relationship	16 (53.3%)	7 (38.9%)	9 (75%)	
**HIV co-infection**				0.807
Yes	17 (56.7%)	11 (61.1%)	6 (50.0%)	
No	10 (33.3%)	6 (33.3%)	4 (33.3%)	
Unknown	3 (10.0%)	1 (5.6%)	2 (16.7%)	
**TB relapse**				0.261
Yes	21 (70.0%)	14 (77.8%)	7 (58.3%)	
No	9 (30.0%)	4 (22.2%)	5 (41.7%)	
**Duration treatment (weeks)**				0.402
*Mean (SD)*	9.5 (8.97)	8.4 (8.39)	11.2 (9.92)	
**Knowledge of TB**				0.757
None	19 (63.3%)	11 (61.1%)	8 (66.7%)	
Some	11 (36.7%)	7 (38.9%)	4 (33.3%)	

Responding to the three stigma-related interview questions and/or elaborating in free text answers, 138 TB patients reported positive or negative perceptions or attitudes regarding TB whereas 162 did not. Socio-demographic characteristics and tuberculosis-related parameters were similar in both populations with the exception of age and educational attainment that was observed to be higher in the first group (with a minor difference of one school year and age difference of three years). Of those, 16/138 patients (12.0%) were younger than 20 years of age. We focused on the group reporting perceptions or attitudes regarding TB (N = 138).

In total, 113/138 TB patients (81.9%) reported that they personally encountered consequences of stigma of which 47/113 patients (41.6%) were female and 13/113 patients (11.5%) were aged under 20 years (Table 3). Using the above-mentioned subcategories, 22/113 stigmatised TB patients (19.5%) reported *experienced stigma*, 61/113 (54.0%) faced *anticipated stigma* and 57/113 (50.4%) had *internalised stigma* (Table 3). The study sample (N = 30) included more women suffering from stigma.

**Table 3:** Socio-demographic characteristics and tuberculosis-related parameters of TBAC study participants who reported TB-related perceptions/attitudes, Lusaka, Zambia.

Variable	All	Stigma				No Stigma	P-value
	N = 138 (%)	N = 113 (%)				N = 25 (%)	Stigma—No stigma
		All stigma	Experienced stigma	Anticipated stigma	Internalised stigma		
		N = 113	N = 22	N = 61	N = 57		
**Sex**							**0.010**
Male	88 (63.8%)	66 (58.4%)	10 (45.5%)	36 (59.0%)	37 (64.9%)	22 (88.0%)	
Female	50 (36.2%)	47 (41.6%)	12 (54.5%)	25 (41.0%)	20 (35.1%)	3 (12%)	
**Age (yrs)**							0.156
*Mean (SD)*	31.8 (11.15)	31.1 (10.27)	33.0 (9.50)	30.6 (9.87)	30.2 (9.96)	34.6 (14.36)	
**Level of education**							0.406
Low (<8 yrs)	60 (43.5%)	51 (45.1%)	8 (36.4%)	30 (49.2%)	24 (42.1%)	9 (36.0%)	
High (≥8 yrs)	78 (56.5%)	62 (54.9%)	14 (63.6%)	31 (50.8%)	33 (57.9%)	16 (64.0%)	
**Marital Status**							**0.083**
Single	61 (44.2%)	46 (40.7%)	12 (54.5%)	19 (31.1%)	20 (35.1%)	15 (60.0%)	
Relationship	77 (55.8%)	67 (59.3%)	10 (45.5%)	42 (68.9%)	37 (64.9%)	10 (40.0%)	
**HIV co-infection**							0.867
Yes	73 (52.9%)	60 (53.1%)	13 (59.1%)	33 (54.1%)	24 (42.1%)	13 (52.0%)	
No	54 (39.1%)	45 (39.8%)	8 (36.4%)	22 (36.1%)	31 (54.4%)	9 (36.0%)	
Unknown	11 (8.0%)	8 (7.1%)	1 (4.5%)	6 (9.8%)	2 (3.5%)	3 (21.0%)	
**TB-relapse**							**0.085**
Yes	36 (26.1%)	26 (23.0%)	5 (22.1%)	13 (21.3%)	13 (22.8%)	10 (40.0%)	
No	102 (73.9%)	87 (77.0%)	17 (77.3%)	48 (78.7%)	44 (77.2%)	15 (60.0%)	
**Duration of treatment (weeks)**							0.312
*Mean (SD*	10.0 (7.949)	9.7 (7.480)	12.9 (7.383)	9.0 (7.216)	8.5 (6.625)	11.5 (9.845)	
**Knowledge of TB**							0.405
None	82 (59.4%)	69 (61.1%)	11 (50.0%)	42 (68.9%)	36 (63.2%)	13 (52.0%)	
Some	56 (40.6%)	44 (38.9%)	11 (50.0%)	19 (31.1%)	21 (36.8%)	12 (48.0%)	

Univariate analyses identified an important difference in sex between stigmatized and non-stigmatized patients. Moreover, relapse cases and married persons were less stigmatized (Table 3). In a multivariate logistic regression model, the variable sex (female) was found to be a significant predictor for stigma (Table 4), and being single or a relapse case were further, albeit non-significant, predictors for stigma. In the sample (N = 30), the variables sex and marital status were also found to have an association with stigma (p<0.1) (Table 2).

**Table 4:** Multivariate logistic regression analyses predicting stigma in 138 TB patients, TBAC study, Lusaka, Zambia.

Variable	B (SE)	OR	95% CI	P-value
**Sex** (female)	1.701 (0.658)	5.479	1.51–19.88	**0.010**
**Marital status** (single)	−0.848 (0.472)	0.428	0.17–1.08	0.073
**TB relapse** (yes)	−0.77 (0.488)	0.462	0.18–1.20	0.113

### Patients’ experience of the TB program

At Kanyama clinic, most patients were satisfied with the TB department (*the ‘TB corner’*) and its staff. However, TB patients described more uncomfortable situations at the general clinic:

*I came to the clinic*, *because I was not feeling well*. *[…] The nurse did not respond well*, *because they suspected TB*. *When I came I was coughing and she was very rude*. *She shouted*: “*If you are coughing*, *this is not the right place to come*. *Go to the TB corner*!” *I felt stigmatized at that first day* (TB patient during FGD).

Several patients were critical about the stigma-reducing measures at the TB corner. They described being too ill to comprehend the one-on-one introduction talk about TB. Other patients explained their treatment supporter was unmotivated, because he/she was not always present at the clinic, did not visit them at home for support or family sensitization, or did not properly answer their questions. Some patients had not known that there was a counsellor present at the clinic. More than a quarter of all patients (28.3%) declared they had not received any sensitization at the clinic.

### TB perceptions

Most patients (94.9%) used biomedical explanations for contracting TB referring to coughing or the airborne nature of TB. However, 112/138 (81.2%) combined this with alternative aetiological explanations such as: sharing cups; familial inheritance; drinking spirits; smoking cigarettes; promiscuous behaviour; abortion; sleeping with a menstruating woman or a woman who had just aborted; a woman adding salt to food while menstruating or after an abortion; or evil spirits. Patients’ aetiological reasoning often blended different supposed causes:

*It feels so bad to have TB*. *And I don't understand*, *because I am so young*. *I am not drinking*, *not smoking*, *I don't have a relationship*. *I am just a student*. *Why do I have TB*? (In-depth interview Claire, 14 year old TB patient).

Claire’s answer illustrates existing negative perceptions regarding TB aetiology and demonstrates her struggle as a TB patient. Additionally, Claire explained that TB-affected children faced a lack of understanding and uneasiness in their social environment, because people generally believed children were unable to contract TB. For this reason, being young was an extra burden for her aggravating TB-related stigma.

The fear for TB was also reflected in frequent use of the term “*Kanayaka*” meaning “*the red light that never switches off*”. This stigmatising term was initially used in the community for HIV patients and a warning to avoid contact. However, we found that this label was also used for TB patients. Some patients and many of their relatives/neighbours believed that HIV and TB were the same disease or that TB patients were always co-infected with HIV. Accordingly, negative attributes associated with HIV, such as (presumed) immorality and promiscuous behaviour, were also attributed to TB patients. Moreover, the term kanayaka foreshadowed the alleged upcoming death of a HIV and/or TB patient. Indeed, various TB patients (28/138, 20.3%) reported encountering these negative attitudes and were consequently approached as if they were ‘doomed to die’. Overall, this linking with HIV and community-based fear and aversion aggravated TB stigma and often prompted patients not to disclose their TB status.

### Experienced stigma

We assigned TB patients to the sub-category *experienced stigma* if they declared being treated differently by relatives/neighbours/friends after disclosure of TB, for instance by facing ridicule, insulting remarks, discrimination, social exclusion, and/or isolation. Social exclusion was often triggered by the idea that TB is highly infectious, manifesting in dining and sleeping separately; avoidance of sexual intercourse; exclusion from activities in school and/or at work. The story of a child with open-TB exemplified such social exclusion prompted by fear of presumed contagiousness:

*After disclosure they tried to avoid her [Helen]*, *run away*, *not even greet*. *Children in school were not allowed to play with Helen because their parents would tell them Helen had TB and they should keep their distance* (In-depth interview Rosemary, mother of 9 year old TB patient Helen).

With the term social isolation, we refer to more drastic social consequences caused by stigmatizing actors, such as divorce, permanent dismissal at work, or ostracism. Various respondents mentioned that some TB patients were banished to a village to live with relatives. The main explanation given for this banishment was to prevent infection of household members and/or to hide patients from neighbours and relatives. This theme recurred during FGDs during which various respondents indicated that women were more often expelled to the countryside than men.

### Anticipated stigma

We assigned the label *anticipated stigma* to respondents who mentioned difficulties disclosing their TB status due to the fear of negative reactions by others.

*I did not tell anybody [..] Because there is too much stigma*. *I mean there is too much fear*. *That is why I used to hide*. *They think if someone is infected with TB*, *he can have any disease*, *he is stupid*, *he is not thinking*. *They spread it to other people*, *and people just add some diseases on top of that*. *Instead of praying*, *they make it worse* (In-depth interview Bo, TB patient).

As this quote illustrates, the social standing of TB patients is negatively affected because people consider them irresponsible and likely to spread TB or other diseases (such as HIV). Faced with prejudices, these patients often concealed their TB in order to avoid insulting remarks, misunderstandings, and a disrupted social status. This is shown by the following quote:

*When you have TB*, *you are degrading yourself if you tell others [that you have TB]*. *You get a problem*. *They will use it against you*. *[…] I did not tell anyone in church*, *because I don’t want to destroy my [social] position there* (In-depth interview Alex, TB patient).

### Internalised stigma

Half of the stigmatised TB patients (50.4%) had internalized the stigmatizing ideas and, consequently, they believed that they were less worthy than others. This belief was expressed by either fear, shame, hopelessness, guilt and/or a loss of self-esteem. Moreover, internalization of devaluating beliefs altered TB patients’ expectations of life.

*I don’t want people to know I have TB*. *To find a husband is difficult*. *Who wants to have us [herself and her sister]*? *We have TB*, *no one will be interested* (In-depth interview Virginia, TB patient).

Virginia and her divorced sister both suffered from TB and had lost hope to ever get (re)married. As they explained elsewhere in the interview, the inability to find a marriage partner severely constrained their social and economic prospects and, thereby, the hope of escaping the severe poverty in which she, her sister and her three children were living.

### Impact of stigma on the TB program

Processes of stigmatization can lead to denying a positive TB diagnosis, non-disclosure, fear, and poor quality of life. Some patients did not want to be seen in the TB corner, were reluctant to openly take tablets, and avoided to be associated with the clinic. As a result, stigma led to patients’ hospital delay and poor treatment compliance and undermined efforts to screen for TB in the households of TB patients. An example hereof was raised during an FGD:

*The nephew of my neighbour got the diagnosis TB at the clinic*, *this means they will do a household screening*, *but the family refused*. *The aunt said*: “*no one can have TB*, *because I believe in God”*, *even though the nephew is smear-positive*. *Instead of testing*, *they do nothing*. *The nephew now has to sleep alone*, *eat alone and no one talks to him*. *He is taking treatment on his own* (TB patient during FGD).

The aunt’s religiously framed argument as to why her nephew could not have a positive TB diagnosis shows how stigma can adversely affect a TB control program. Increasingly deprived of social support, the nephew was socially excluded, hindering his compliance with treatment guidelines. Moreover, since household members believed that God protected them against TB, their rejection of TB screening could possibly delay diagnosis and fuel the spread of TB. This link between stigmatization and the TB control program is also present when TB patients were banished to their relatives’ village:

*I did not finish my treatment*, *because after the hospital my mother and grandmother took me to the farm [in the village]*. *It was a long distance to the hospital*. *I ran out of TB drugs and I didn't have transport*. *I couldn't walk and my mother got tired of it*. *Four years later I came from the farm here [at the clinic]* (In-depth interview Sarah, TB patient).

Sarah’s social position was not only adversely influenced, she was also sent far away by her family. This banishment and the inadequate availability or accessibility of TB treatment in the villages led to poor treatment compliance and, in Sarah’s case, a sharp decline of her health.

### Shortcomings of sensitization programs

During the (structured) interviews and FGDs, the majority of respondents emphasised that inadequate biomedical knowledge and existing misconceptions of TB among community-members were key factors in negative attitudes and behaviour toward TB patients. Some respondents educated relatives/neighbours, such as the earlier mentioned Rosemary:

*When I figured out that children in school were told by their parents to keep [their] distance from Helen [her daughter]*, *I got mad*. *I explained all the parents that Helen had been on treatment since more than two weeks and that she was not infectious anymore*. *Then the mothers and children apologized*. *That helped*. *Now the situation is fine* (In-depth interview Rosemary, mother of TB patient).

In this case, providing biomedical knowledge reduced fear and stigma. However, not all respondents were aware of those misconceptions and/or able to (successfully) confront their stigmatizing environment with biomedical information in order to change these attitudes. Despite existing sensitization programs, various health care providers and TB patients described difficulties in reaching all TB patients and community members, and in making the TB-information understandable to patients.

*Stigma can kill a lot of people*. *[…]They are not encouraged to seek health care. [..] Sensitization is working*, *but it’s a matter of listening*. *People will hear*, *but not listen or understand*. *There should be more active sensitization*, *get them involved*, *so they listen and understand* (In-depth interview Alex, TB patient).

## Discussion

Based on a mixed methods design, this study aimed at assessing stigma for TB patients in Lusaka, Zambia. We focused on TB-related stigmatizing perceptions and attitudes mentioned by the TB health care workers and 138 TB patients of the TBAC study including their influence on patients' lives and the TB control program. In total, 82% of these patients were affected by consequences of stigma.

Some of the stigma-related TB perceptions found in this study were likewise mentioned in studies in other parts of the world: the assumption that TB patients are careless and responsible for their own infection [19]; the association of TB with HIV [12] and with immoral behaviour [19]; and perceptions that TB is incurable [4] and very infectious throughout the treatment trajectory [17]. These perceptions were often associated with patients’ fear of disclosure, discrimination, social exclusion, and/or isolation [12,17,19,27].

Within community-level discourses, perceptions of TB were often linked to HIV, a finding that coincides with a Zambian study on HIV-TB related stigma [12]. We found that the derogatory term *Kanayaka*, used to warn against contacting with HIV patients [33], was also used for TB patients. One Zambian HIV-study briefly referred to the usage of this term for contagious disease in general [34] explaining that HIV patients faced an extra dimension of stigma as the term additionally symbolised their upcoming and inevitable death. However, we found that Kanayaka for TB patients was used in a similar stigmatizing manner, labelling them both as a source of infection and as doomed to death. Additionally, TB patients experienced the negative attributes of HIV, such as allegations of immoral behaviour. The linking with HIV seriously aggravated TB stigma and illustrates that research on TB should not ignore HIV.

In contrast to findings in a Nepalese and a Zambian study [27,35], respondents did not associate TB with poverty or low class. A plausible explanation is the relatively limited socio-economic variation in the studied population. This resonates with World Bank statistics [36] that 60.5% of the Zambian population lives under the poverty line and a socio-economic case study of Lusaka describing that poverty levels are specifically high in slum areas such as Kanyama [37].

Two groups that have proven extra vulnerable were children and women with TB. First of all, childhood TB is a recognised, yet under-researched problem [38–40] and studies on children and TB-related stigma are scarce [41,42]. Our quantitative data demonstrated that children were as vulnerable as adults to suffer from the social consequences of stigma. During qualitative data collection, several patients explained that community members generally thought children were unable to contract TB. A paradoxical finding was that as a result, TB infected children faced an extra dimension of stigma being confronted with misunderstanding and uneasiness.

Secondly, quantitative analysis showed that women were significantly more vulnerable to stigma than men. Additionally, qualitative analysis showed more women faced stigma, despite the higher number of men interviewed. This finding resonates with previous research worldwide [17,43–45]. Another study conducted in Lusaka describes the vulnerable position of female TB patients explaining that this group has more often diagnostic delays because of stigma [46]. Moreover, we found that women are often blamed in the local understandings of TB transmission, a finding that parallels a study on HIV-related stigma in Zambia [33] in which women are blamed for the spread of HIV and more impacted by stigma. Gender inequality enhances the vulnerability for stigma and, additionally, leads to different consequences of stigma among women and men [33,35,43–45]. Following this argument, the differences in stigma between male and female patients reflect gender inequalities in Zambian society where, historically, women often have more limited rights and power than men [47–50]. In this context, TB-related stigma can be perceived as being rooted in cultural patterns of gender inequality. Therefore, we postulate that there is an important link between stigma, gender, and TB perceptions, a connection insufficiently recognised in the literature on TB-related stigma as in TB policies.

It is important to fight stigma as its social consequences hinder effective TB control causing delayed diagnosis and poor treatment compliance. Consequently, this leads to poor treatment outcomes and treatment failure, fuels ongoing transmission, and facilitates the emergence of TB drug resistance. Kanyama clinic has acknowledged the importance of fighting stigma, and developed several interventions such as a treatment supporter program, family and community sensitization, and counselling. However, based on the findings of this study these programs are not (yet) functioning optimally.

Many patients reported experienced and/or anticipated stigma. Anticipated stigma should be taken as seriously as experienced stigma as it reflects the prevalence of stigmatising understandings and practices in the community. Accordingly, respondents mentioned repeatedly that community members possessed insufficient (biomedical) knowledge and hold TB misconceptions. However, ascribing stigma solely to a knowledge deficit and assuming that knowledgeable people will not stigmatize ignores the cultural context with deep-seated beliefs [51]. Therefore, instead of simply relaying biomedical knowledge, we advocate interactive sensitisation programs at schools and in the community that stimulate discussion and raise awareness regarding stigma. In addition, stigma-reducing measures should not only be implemented at the TB corner, but across the clinic.

Strikingly, almost one third of patients reported that they never received TB-information, notwithstanding the available sensitization programs at the clinic and the skilful, experienced staff. This gap was only partially explained by patients who mentioned that they had been too ill to pay attention; that educational sessions did not sufficiently capture patients’ attention; and that information was often too complicated for patients to comprehend.

Patients’ ignorance regarding TB can be associated with the fact that about half of the stigmatized patients had internalized stigmatizing beliefs. They reported shame or self-exclusion indicating that, notwithstanding education and supervision, they blamed themselves for contracting TB and lacked sufficient ability to ignore or resist stigmatizing cultural ideas. To diminish internalised stigma, more empowerment of patients with regards to their TB status is needed, either through sensitization, counselling, or support groups. For instance, the clinic’s current running and successful support groups for TB-HIV co-infected patients should extend to all TB patients regardless of their HIV status.

Lastly, within these programs more attention is needed for the vulnerable position of women and children. Since women disproportionally bear the burden of TBstigma in Zambia, they urgently need better care. Although gender inequality is influenced by structural patterns that cannot be changed easily, it is important to take these factors into consideration. Interventions aimed at combating TB and TB-stigma need to acknowledge that women often have a lower social status, insecure economic position, and receive less education. Furthermore, children may be even more affected by stigma than adults that calls for interventions specifically targeted at this vulnerable group.

### Limitations and strengths

The study was embedded into the larger TBAC-study allowing identifying those 138 patients who described positive or negative TB-related perceptions and attitudes for detailed assessment. However, it may be that we underestimated the extent of stigma: the structured interview questions might have been inadequate to provoke associated answers or shame resulting from stigma could have made respondents reluctant to discuss the topics. As we did not find major differences in characteristics between the two groups, we consider risk of selection bias to be minor. In future research, we suggest that the quantitative component of the study should contain more stigma-related questions to allow a focus on the overall study group. Additionally, during qualitative research, we found that children and adolescents faced an extra dimension of stigma, yet their low representation within the study group impeded extensive analysis. Further research is required to study TB-related stigma for this group in more detail. Furthermore, the fact that patients referred to rural areas were lost to follow up, reflects the poor administration systems in rural clinics. In addition, some patients referred to rural clinics' difficult accessibility and availability, calling for a similar research in a rural area. Unfortunately, we did not document patients' non-response rate.

The major strength of this study was the mixed methods design enabling triangulation of study findings. Quantitative research illustrated how many patients struggled with TB-related stigma and identified sex to be significantly associated with stigma. Subsequently, qualitative data analysis was conducted to contradict or confirm quantitative outcomes, to explain the statistical relationship, and to provide in-depth case illustrations for a comprehensive understanding of TB-related stigma.

## Conclusion

Despite the existence of various programs fighting TB-related stigma in urban Zambia, TB patients continue to experience stigma extensively. Prominent findings are the high vulnerability of women to stigma, the prevalence of stigma among children, the influence of stigma-related issues on the TB control program, and the stigma-provoking misconceptions in the community regarding TB transmission, the relation between TB and HIV, and the perceived upcoming death of TB patients. We therefore recommend a revision of both the content and the implementation of interventions aimed at reducing stigma.

## Supporting Information

S1 Fig Research methods & study population TBAC study, Lusaka, Zambia. (TIF)
